# A Comparative Study on the Efficiency and Sustainability of Rice Bran Oil Extraction Methods

**DOI:** 10.3390/foods14234076

**Published:** 2025-11-27

**Authors:** Lucia Sportiello, Maria Concetta Tenuta, Roberta Tolve, Fabio Favati, Gabriele Quarati, Giovanna Ferrentino

**Affiliations:** 1 Department of Biotechnology, University of Verona, Strada Le Grazie 15, 37134 Verona, Italy; lucia.sportiello@univr.it (L.S.); gabriele.quarati@univr.it (G.Q.); 2 Faculty of Agricultural, Environmental and Food Sciences, Free University of Bolzano, Piazza Università 1, 39100 Bolzano, Italy; mariaconcetta.tenuta@unibz.it (M.C.T.); giovanna.ferrentino@unibz.it (G.F.)

**Keywords:** rice bran oil, by-products valorization, green extraction, life cycle assessment

## Abstract

Rice bran, a rice milling by-product, is a rich source of bioactives such as tocopherols and γ-oryzanol, with promising antioxidant properties. This study compared three extraction techniques—Soxhlet, maceration, and supercritical CO_2_ (SC-CO_2_)—to identify the method offering the best balance of rice bran oil (RBO) recovery, composition, and sustainability. Although all methods yielded similar oil quantities (~9.5–10.8%), SC-CO_2_ extraction achieved superior preservation of bioactives, with the highest tocopherol (116.9 µg/g) and γ-oryzanol (13.2 mg/g) levels. Antioxidant capacity, assessed via FRAP, ABTS, and DPPH assays, was consistently higher in SC-CO_2_-extracted oil. The fatty acid profile further confirmed the advantages of SC-CO_2_ extraction, with the oil showing a high proportion of unsaturated fatty acids (86.3%) and low saturated content (13.6%). In contrast, Soxhlet- and maceration-extracted oils contained higher saturated fractions (56.5% and 60.1%, respectively) and lower unsaturated content, reflecting the impact of thermal and solvent exposure on the lipid composition. Environmental impacts were quantified through cradle-to-gate life cycle assessment (LCA), showing that SC-CO_2_ extraction led to the lowest ecological burden due to its solvent-free process and lower energy demand. Normalizing impacts on both oil yield and bioactive content further highlighted its advantages. These findings place SC-CO_2_ extraction as a green, efficient alternative for valorizing rice bran, yielding a high-quality, antioxidant-rich oil suitable for food and cosmetic applications. The integrated chemical and environmental evaluation underscores the potential for a sustainable bioeconomy, effectively turning agricultural residue into functional ingredients.

## 1. Introduction

With an estimated global production of approximately 520 million tonnes in 2023/24, rice (*Oryza sativa* L.) is one of the most widely cultivated and consumed staple cereals, serving as a fundamental food source for more than half of the world’s population. Its production underpins food security, supports millions of livelihoods, and represents a key component of worldwide agricultural economies [1].

Alongside its critical role in human nutrition, rice processing generates large amounts of by-products, the most abundant of which is rice bran. This outer layer of the grain, removed during polishing, constitutes approximately 10–15% of the total grain weight, with worldwide production exceeding 76 million tons annually [2,3,4]. Traditionally considered a low-value by-product, rice bran is used for animal feeding or discarded. After milling, the rapid activation of endogenous lipases promotes triglyceride hydrolysis into free fatty acids, leading to rancidity and limiting direct applications [5]. Despite these drawbacks, rice bran is increasingly recognized as a reservoir of valuable bioactive compounds with nutritional, functional, and economic value. Its valorization not only prevents waste but also opens the way for developing high-value ingredients for the food, nutraceutical, pharmaceutical, and cosmetic industries, aligning with sustainability and circular economy principles [6,7,8].

Among rice bran derivatives, rice bran oil (RBO) has drawn notable attention due to its unique profile of phytochemicals, including tocopherols, tocotrienols, phytosterols, polyphenols, flavonoids, and especially γ-oryzanol. This latter fraction, initially thought to be a single compound, is a mixture of ferulic acid esters of triterpenoids and phytosterols, with cycloartenyl ferulate, 24-methylenecycloartanyl ferulate, and campesteryl ferulate as the major constituents [3,9].

Extraction methodology plays a critical role not only in determining RBO yield but also in preserving the integrity of bioactive compounds. Conventional methods, such as Soxhlet and maceration with hexane, provide good recovery but involve high temperatures, solvents, and safety concerns [10,11]. Hexane-based extractions, including Soxhlet, frequently achieve effective oil yields. For instance, Wongwaiwech et al. [12] reported a hexane extraction yield of 26%. However, this approach has been associated with the production of RBO exhibiting a darker color and elevated free fatty acid content. Consistently, Zhao et al. [13] observed similar results comparing the RBO obtained via hexane extraction to those obtained using supercritical CO_2_ (SC-CO_2_). Additional studies have indicated that prolonged exposure to heat and organic solvents can lead to the degradation of heat- and solvent-sensitive bioactive compounds, including γ-oryzanol and tocopherols [14,15].

Alternative solvents, including d-limonene and carbon dioxide-expanded hexane, have been explored to improve both the safety and selectivity of lipid extraction. Mamidipally and Liu [16,17] reported that d-limonene afforded yields nearly equivalent to those obtained with hexane, while Okajima et al. [18] achieved high extraction efficiencies of 24.0–25.7% using CO_2_-expanded hexane. Cold-press extraction, performed at ambient temperatures, typically results in lower oil yields compared to solvent-based methods (3.2–6.2%) but preserves higher levels of bioactive compounds and antioxidant activity, making it suitable for applications emphasizing functional and nutritional properties [12,19]. Other innovative approaches, such as subcritical dimethyl ether and enzyme-assisted extractions, have demonstrated the potential to achieve a favorable balance between yield, bioactive retention, and sustainability, with reported γ-oryzanol and phytosterol contents of 12.14 mg/g and 17.84 mg/g, respectively [12].

Furthermore, RBO can be efficiently and sustainably extracted using SC-CO_2_ technology, which operates at moderate temperatures (40–70 °C) and high pressures (150–500 bar), allowing the selective recovery of antioxidants without leaving solvent residues. Reported extraction yields can reach values comparable to those achieved with hexane in some studies. Under optimal conditions (500 bar, 60 °C, 1.5 h), Balachandran et al. [20] obtained a maximum yield of 22.5%, along with γ-oryzanol concentrations of 5.8–11.11 mg/g and phytosterol levels of 15.35–19.12 mg/g, while Mingyai et al. [19] reported superior antioxidant activity and color retention in black RBO. In addition, SC-CO_2_ consistently produces oil with a lighter color, lower free acidity, and reduced phosphorus and iron contamination compared to hexane extraction.

Despite differences in efficiency, bioactive retention, and oil quality, technological performance alone does not fully capture environmental sustainability. Energy consumption, solvent use, and overall process efficiency strongly influence the ecological footprint. Life Cycle Assessment (LCA) provides a systematic framework for quantifying environmental burdens and comparing conventional with emerging extraction methods [21,22]. Crucially, LCA studies must define the system boundary to include key impact categories, such as Global Warming Potential (GWP), abiotic resource depletion, and human/eco-toxicity potential, which addresses the hazardous waste associated with traditional organic solvents like n-hexane [23].

Recent comparative analyses highlighted a critical trade-off: while green approaches, such as Supercritical Fluid Extraction (SFE) or Enzyme-Assisted Extraction (EAE), drastically reduce chemical inputs and eliminate the need for hazardous solvent disposal associated with traditional extraction methods, they often exhibit higher energy demands due to the necessity of high pressure and temperature operation [21,24]. This increased consumption of non-renewable energy frequently leads to a greater overall GWP, which can substantially offset the environmental benefits gained from solvent elimination [21,25]. A further LCA on green extraction of rosemary antioxidants confirmed that, despite eliminating solvents, methods like SFE and Pressurized Hot Water Extraction can have comparable or higher environmental impacts due to their high energy demands for pressure and heating control [26]. Furthermore, the selection of the functional unit is pivotal to ensure that sustainability metrics are aligned with commercial and nutritional value [22,27,28].

In this study, RBO was extracted using three extraction methods—Soxhlet, maceration, and SC-CO_2_—and characterized in terms of yield, acidity, value peroxide value (PV), fatty acids (FA) profile, total phenolic content (TPC), total flavonoid content (TFC), antioxidant activity (FRAP, ABTS, DPPH), and the contents of α-tocopherol and γ-oryzanol. Additionally, the environmental impacts of each extraction method were assessed through a cradle-to-gate LCA, integrating sustainability considerations into the valorization of this by-product. To date, no previous studies have combined detailed chemical characterization with LCA of different RBO extraction methods. By combining chemical, functional, and environmental analyses, this work provides a comprehensive evaluation of RBO as a high-value antioxidant source, demonstrating its potential for economically and ecologically sustainable applications in the food and cosmetic sectors.

## 2. Materials and Methods

### 2.1. Chemicals and Materials

All solvents and reagents used in this study were of analytical or HPLC grade and were obtained from Sigma-Aldrich (Saint Louis, MO, USA). Milli-Q water was used throughout the analyses. Rice bran used for oil extraction was purchased from Riseria Melotti (Isola della Scala, Verona, Italy). Upon delivery, the bran was pre-treated at 100 °C for 15 min to inactivate endogenous lipase activity and stored in airtight containers under controlled temperature conditions (at −20 °C) and protected from light to ensure the stability of bioactive compounds.

### 2.2. Extraction of Rice Bran Oil

Rice bran oil was extracted using three different extraction methods: E1 (Soxhlet extraction), E2 (maceration), and E3 (SC-CO_2_). Extractions were performed within two weeks after the lipase inactivation pre-treatment to prevent oxidation. All procedures were carried out in triplicate using independent biological replicates. After each extraction, the resulting oil samples were stored in amber vials, flushed with nitrogen and stored at −80 °C for further analysis.

#### 2.2.1. Soxhlet Extraction

Soxhlet extraction was performed by placing approximately 25 g of rice bran into a cellulose thimble with 20 g of glass beads (Ø 3.0 mm) to increase surface area. The thimble was sealed with glass wool and inserted into a Soxhlet apparatus, mounted above a flask containing 150 mL of hexane, connected to a condenser fed with cooling water. The system was heated to the boiling point of the solvent. The extraction lasted for 3 h. Afterwards, the mixture contained in the flask was connected to a rotary evaporator (Rotavapor Buchi R-205, Buchi Labortechnik AG, Flawil, Switzerland) and the solvent was evaporated. A vacuum pump was used to accelerate the process.

#### 2.2.2. Maceration

Maceration was conducted according to the method described by Ghasemzadeh et al. [29]. Briefly, 5 g of rice bran was mixed with 50 mL of hexane. The mixture was stirred in the dark for 2 h, then centrifuged at 8228× *g* for 10 min at 20 °C (Allegra X-22R, Beckman Coulter, Brea, CA, USA). As reported above, the organic phase was recovered and evaporated to obtain the oil.

#### 2.2.3. Supercritical CO_2_ Extraction

SC-CO_2_ extraction was carried out following the protocol of Ferrentino et al. [30]. One hundred g of rice bran was loaded into a 1 L stainless steel extraction vessel of a semi-batch pilot unit equipped with two gravitational separators (Superfluidi S.R.L., Padova, Italy). The extraction was conducted at 40 °C and 220 bar with a CO_2_ flow rate of 1 L h^−1^ CO_2_ for 180 min. Upon completion, the extracted oil was collected from the first separator.

### 2.3. Rice Bran Oil Characterization

#### 2.3.1. Yield

Oil yields from each extraction procedure were determined to evaluate the efficiency of the different methods applied. The yield was calculated as the percentage of the ratio between the recovered rice bran oil (*RBO*) and the initial weight of the rice bran (*RB*) used for each extraction. The following equation (Equation (1)) was used:

(1) $$Y\mathrm{i}\mathrm{e}ld\;\left(\%\right)=\frac{RBO\left(g\right)}{RB\left(g\right)}\times 100$$

where *RBO* is the mass of oil extracted, and *RB* is the mass of the rice bran sample subjected to extraction.

#### 2.3.2. Acidity and Peroxide Value

The acidity value (AV) content of rice bran oil (RBO) samples obtained from the different extraction methods was determined following the AOCS Official Method Ca 5a-40 [31]. For each analysis, 2.5 g of oil were dissolved in a diethyl ether–ethanol mixture (1:1 (*v*/*v*)), and 0.3 mL of phenolphthalein was added as a visual indicator. The liberated fatty acids were then titrated with 0.05 M NaOH until the equivalence point was reached. The FFAs’ value was calculated and expressed as a percentage of oleic acid equivalents. To enhance accuracy, the results were adjusted using Equation (2), as proposed by Krishna et al. [32], which considered the influence of γ-oryzanol:

(2) real AV=observed AV−(% oryzanol in the oil)·0.425

The peroxide value (PV), an indicator of primary lipid oxidation, was determined according to AOAC Official Method 965.33 [33]. Briefly, 2 g of oil were dissolved in a mixture of chloroform and acetic acid (2:3 (*v*/*v*)), and a saturated KI solution was added. After standing in the dark for 5 min, the mixture was treated with 30 mL of water and a few drops of 1% starch solution. The liberated iodine was then titrated with standardized 0.01 N sodium thiosulfate solution until the blue color disappeared. Results were expressed as milliequivalents of active oxygen per kilogram of oil (mEq O_2_/kg).

#### 2.3.3. Fatty Acid Profile Determination

The fatty acid composition of RBO samples oils was determined using Gas Chromatography–Time-of-Flight Mass Spectrometry (GC-ToF/MS). Fatty acids were first extracted and converted into their corresponding methyl esters (FAMEs). Briefly, 200 mg of oil were mixed with 8 mL of hexane and 300 µL of 2 M KOH in methanol. The mixture was vortexed and allowed to stand until a clear phase separation occurred. The upper hexane layer containing the FAMEs was then collected and transferred into GC vials for analysis. Separation was carried out on a MEGA-Wax capillary column (60 m × 0.25 mm × 0.25 µm) using helium as the carrier gas at a flow rate of 1 mL/min. The injector and transfer line temperatures were set at 275 °C and 270 °C, respectively, with the oven temperature programmed from 125 °C to 270 °C at 4 °C/min.

Mass spectra were recorded in electron ionization mode (70 eV) across an *m*/*z* range of 35–530. Identification of compounds was achieved by comparison of retention indices and mass spectra with standard FAME mixtures and the NIST 2017 library [34], and quantification was based on the integration of deconvoluted chromatographic peaks using ChromaToF software (version 4.20, LECO Corporation, St. Joseph, MI, USA).

#### 2.3.4. Polyphenols, Flavonoids and Antioxidant Activity Determinations

For all determinations, 0.1 g of each oil sample was diluted in 5 mL of ethanol to obtain a final concentration of 20 mg/mL. The solutions were filtered through 0.45 µm PTFE syringe filters prior to spectrophotometric analysis.

Total Phenolic Content (TPC) was performed according to the modified method of Singleton et al. [35]. Briefly, 200 µL of ethanolic extract was mixed with 200 µL of diluted Folin–Ciocalteu reagent (0.2 M), incubated in the dark for 10 min, followed by the addition of 4 mL saturated Na_2_CO_3_ solution (0.7 M) and 5 mL of milliQ water were added. Samples were incubated under shaking for 60 min at room temperature (20 ± 2 °C) and the absorbance was measured at 765 nm using a spectrophotometer (Infinite 200 Pro MPlex, Tecan, Männedorf, Switzerland). Results were expressed as mg gallic acid equivalents per gram of oil (mg GAE/g), based on a calibration curve prepared from gallic acid standard solutions (30–1000 ppm).

Total Flavonoid Content (TFC) was carried out using the method of Gouveia et al. [36] with minor modifications. A 100 µL aliquot of filtered extract was mixed with 440 µL NaNO_2_ (0.066 M), incubated for 5 min at room temperature (20 ± 2 °C), then 60 µL of AlCl_3_ (0.75 M) were added. After 6 min, 400 µL of NaOH (0.5 M) was added and the mixture was shaken for 30 s. The absorbance was measured at 500 nm, and TFC was expressed as mg catechin equivalents per gram of oil (mg CAE/g), using catechin as standard for the calibration curve (30–1000 ppm).

Ferric Reducing Antioxidant Power (FRAP) was evaluated following the method of Benzie and Strain [37]. A 10 µL aliquot of extract was added to 1.8 mL of FRAP reagent—prepared by mixing 300 mM acetate buffer (pH 3.6), 10 mM TPTZ in 40 mM HCl, and 20 mM FeCl_3_ in a 10:1:1 ratio—and 1 mL of milliQ water. After 10 min of incubation at 37 °C, the absorbance was measured at 593 nm. Results were expressed as µmol Trolox equivalents per gram of oil, using Trolox as standard for the calibration curve from 0.0312 to 2 mM.

2,2′-Azino-bis(3-ethylbenzothiazoline-6-sulfonic acid) assay (ABTS) was conducted according to Pellegrini et al. [38]. A stock ABTS•^+^ solution was prepared by reacting 7 mM ABTS with 2.45 mM potassium persulfate and incubating it in the dark for 16 h. Prior to use, the solution was diluted to an absorbance of 0.700 ± 0.020 at 734 nm. For the assay, 200 µL of extract was added to 9.8 mL of ABTS•^+^ solution, mixed, and left to react in the dark for 60 min. The absorbance was recorded at 734 nm, and antioxidant activity was expressed as µmol Trolox equivalents per gram of oil.

2,2-Diphenyl-1-picrylhydrazyl radical scavenging activity (DPPH) was measured according to Rotondi et al. [39], with slight modifications. A DPPH solution was prepared by dissolving 19.7 mg of DPPH in 10 mL of methanol and diluting it to an absorbance of 0.44 ± 0.02 at 515 nm. In the assay, 2.9 mL of DPPH solution were mixed with 100 µL of extract and incubated in the dark at room temperature (20 ± 2 °C) for 30 min. The absorbance was measured at 515 nm, and the results were reported as µmol Trolox equivalent per gram of oil.

#### 2.3.5. Tocopherol and γ-Oryzanol Determination

α-Tocopherol content was quantified using the method described by Pocklington and Dieffenbacher [14]. In brief, 0.8 g of oil were dissolved in 10 mL of HPLC-grade hexane and filtered through a 0.45 µm PTFE syringe filter. HPLC analysis was performed under isocratic conditions using an adsorbosil silica column (5 µm, 250 × 4.6 mm, Alltech) with a mobile phase composed of isopropanol/hexane (0.7/99.3 (*v*/*v*)) at a flow rate of 1 mL/min. The eluate was monitored using a fluorescence detector at 290 nm (excitation) and 330 nm (emission). Quantification was carried out using external calibration with α-tocopherol standard in the range of 1.3 to 9.1 ng/µL, and results were expressed as µg α-tocopherol per mg of oil.

γ-Oryzanol content was determined by HPLC with a DAD detector (330 nm), following the method of Benito-Román et al. [40]. Approximately 30 mg of oil were dissolved in 1 mL of 2-propanol, filtered through a 0.45 µm filter, and 10 µL of the solution were injected into a Zorbax XDB C18 column (5 µm, 150 × 4.6 mm). The mobile phase consisted of acetonitrile/methanol/2-propanol (50:40:10 (*v*/*v*/*v*)) at isocratic flow. Quantification was achieved by external calibration using a commercial γ-oryzanol standard, with a concentration range of 15.6–500 mg/L. Results were expressed as mg of γ-oryzanol per gram of oil.

### 2.4. Modeling of Environmental Impact Using LCA

**Figure 1:**
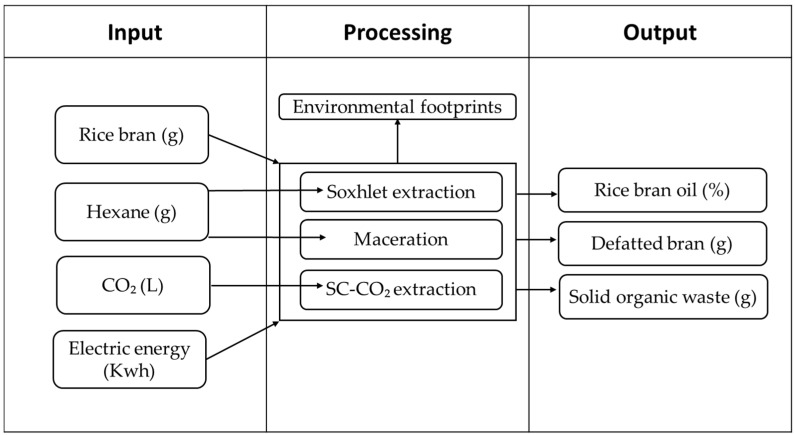
Inputs and outputs of the three extraction methods for the LCA.

The evaluation of the environmental impact of rice bran oil extraction using the three different extraction methods was carried out through a Life Cycle Assessment (LCA) approach with a cradle-to-gate system boundary, i.e., from rice cultivation to the laboratory-scale extraction process. The main goal of this first-phase analysis was to compare the environmental performance of the extraction methods. The functional unit was defined as 10 g of rice bran oil obtained using the supercritical CO_2_ method (Case A). A supplementary assessment was also conducted using an alternative functional unit based on product quality, defined by the amount of α-tocopherol extracted (FU = 1000 µg of α-tocopherol−Case B). The system boundaries of the LCA are presented in Figure 1. For the purpose of this analysis, rice bran was conservatively treated as a by-product rather than a waste, applying mass-based allocation between the main product (rice) and its co-products. The study was performed using OpenLCA software (version 2.5.0, GreenDelta GmbH, Berlin, Germany), with inventory data sourced from Ecoinvent v3.11 and Agribalyse_v301_27052021 databases.

### 2.5. Statistical Analysis

The rice bran oil extractions were performed in triplicate. The results were expressed as mean values with standard deviation. Statistical analysis was performed using one-way ANOVA to evaluate significant differences among the measured variables. Tukey’s post hoc test was applied to assess the significance of pairwise comparisons (*p* < 0.05). All statistical analyses were carried out using XLSTAT software (Premium version 2019.4.2, Addilnsoft, SARL, Paris, France).

## 3. Results

### 3.1. Rice Bran Oil Characterization

In this study, three different extraction methods were applied to obtain rice bran oil: E1 (Soxhlet extraction), E2 (maceration), and E3 (SC-CO_2_ extraction). Soxhlet and maceration were included to compare continuous and static solvent extraction systems using the same solvent but differing in temperature and energy input. This comparison highlights the effects of thermal and mass-transfer conditions on yield and bioactive stability, providing a baseline for evaluating the advantages of SC-CO_2_ extraction.

The extraction yields were approximately 10.8 ± 1.5% for E1, 9.5 ± 0.7% for E2, and 10.0 ± 1.3% for E3, indicating comparable efficiencies among the techniques tested. However, the actual amount of oil recovered varied notably due to both the extraction kinetics and the equipment setup. Specifically, 3.6 g of oil were obtained after 3 h of Soxhlet extraction, 0.4 g after 2 h of maceration, and 10 g after 1 h 30 min of SC-CO_2_ extraction. In addition, the initial rice bran mass processed per cycle in E1 and E2 was considerably lower than in E3, owing to equipment constraints, further contributing to the lower oil recovery.

Therefore, although the percentage yields appear similar, the smaller absolute recovery for E1 and E2 suggests that these solvent-based extractions did not reach equilibrium, likely due to kinetic and mass-transfer limitations under short and non-optimized laboratory conditions—typical of diffusion-controlled systems. Conversely, the E3 yield is mainly affected by the selective solubility of CO_2_ for neutral triacylglycerols [41,42] and its exclusion of polar compounds and waxes, as reported in previous comparisons with conventional solvents [20,43].

Additionally, the oils were characterized for quality using key indicators of chemical stability, namely acidity value (AV) and peroxide value (PV), together with fatty acid (FA) profiling (Table 1 and Table 2). These parameters collectively provide insights into the oxidative status, thermal degradation, and lipid fractionation associated with each extraction method.

As can be noted, the oil extracted by SC-CO_2_ (RBO-E3) exhibited the highest proportion of total unsaturated fatty acids (UFAs) (~86%), confirming the superior selectivity of this technique toward unsaturated lipid species. Monounsaturated fatty acids (MUFAs) accounted for 55.67% of the total fatty acids (dominated by 55.56% oleic acid), while polyunsaturated fatty acids (PUFAs) represented 30.77% (predominantly 30.62% linoleic acid). Conversely, RBO-E3 contained the lowest proportion of Saturated Fatty Acids (SFAs) (13.56%), consistent with previous findings [44] that demonstrate SC-CO_2_ extraction effectively concentrates oleic- and linoleic-rich triacylglycerols while minimizing the co-extraction of waxes and free saturated components.

This favorable FA profile correlated with the lowest PV (5.84 mEq O_2_/kg) in RBO-E3, demonstrating superior oxidative stability. This finding aligns with previous reports indicating that SC-CO_2_ extraction enhances oxidative stability by minimizing oxygen exposure during processing. However, RBO-E3 recorded the highest AV (60.14% oleic acid equivalent). Given that the bran was stabilized, this elevated acidity suggests that either the stabilization process was incomplete or that the high pressure of the SC-CO_2_ system at moderate temperature significantly increased the solubility and extraction efficiency of the FFAs already present in minor quantities, resulting in a preferentially concentrated extract of FFA compared to E1 and E2 extractions. Despite the high AV, the low PV confirms that the SC-CO_2_ process effectively co-extracted lipophilic antioxidants, which protect the lipid matrix from subsequent oxidation.

In contrast, the oils obtained via maceration (RBO-E2) and Soxhlet extraction (RBO-E1) were characterized by higher proportions of SFAs (60.09% and 56.47%, respectively). In both cases, palmitic acid (C16:0) predominated (48.10% in RBO-E2 and 25.76% in RBO-E1), followed by stearic acid (C18:0). Critically, RBO-E1 showed the highest PV (10.43 mEq O_2_/kg) and contained no detectable PUFAs (0%), with its unsaturated fraction consisting entirely of oleic acid (43.53%). The compositional degradation, coupled with the higher oxidation value, suggests that the extended reflux during Soxhlet extraction can promote oxidation or hydrolysis of unsaturated species [43], potentially explaining the absence of detectable PUFAs and the higher saturated fraction observed. By contrast, solvent-based methods may favor the co-extraction of long-chain saturated compounds and products due to prolonged contact and exposure to elevated temperatures.

The compositional differences can therefore be attributed to the distinct extraction mechanisms. SC-CO_2_ extraction selectively solubilizes neutral triacylglycerols, particularly those rich in unsaturated fatty acids, while excluding heavier waxes and unsaponifiable matter [41]. These findings highlight the superior preservation of unsaturated fatty acids achieved through SC-CO_2_ extraction, supporting its use as an efficient and mild technique for producing high-quality rice bran oil with enhanced nutritional and oxidative stability.

To further assess the nutritional profile and antioxidant potential of the extracted bran oils, total phenolic content (TPC), total flavonoid content (TFC), and antioxidant activities (FRAP, ABTS, and DPPH assays) were evaluated. The outcomes of these analyses, which reflect both the phenolic composition and radical-quenching potential of oils obtained by the three extraction methods (RBO-E1, RBO-E2, and RBO-E3), are presented in Table 3.

These values fall well within, or slightly above, the ranges reported in the recent literature for RBO [12,27,45,46]. TPC values ranged from 1.76 to 2.05 mg GAE/g, which is slightly higher than values typically found in parboiled or solvent-extracted oils and comparable to those obtained through cold-pressing techniques [12]. Likewise, TFC values, ranging from 4.67 to 5.52 mg CE/g, exceeded those commonly reported for conventionally extracted white-bran oils, although they remained below the higher concentrations observed in flavonoid-rich cultivars [27,45].

The radical scavenging activity assays confirmed the antioxidant potential of all three oils. ABTS values ranged from 5.25 to 6.89 mg TE/g, slightly exceeded those reported for standard RBO extracts, while DPPH values (3.01–3.84 mg TE/g) and FRAP activity (3.03–6.22 mg TE/g) also aligned with or outperformed previously published data [27,46]. Among the three extraction methods, RBO-E3, that was obtained via supercritical CO_2_ extraction, consistently exhibited the highest antioxidant values across all assays, indicating superior retention of bioactive compounds.

Importantly, beyond its strong functional and nutritional performance, E3 also offers considerable added value from an environmental sustainability perspective. In contrast to conventional solvent-based methods, supercritical CO_2_ extraction is a green technology that eliminates the use of hazardous organic solvents, enables selective recovery of valuable compounds, and operates under relatively mild conditions that reduce disadvantages such as thermal degradation and energy consumption [47,48]. When combined with its enhanced antioxidant profile, the RBO-E3 extract emerges as the most promising candidate, delivering both high-quality oil and lower environmental impact, in alignment with current trends toward sustainable, clean-label food ingredients.

As a final step in the evaluation of the nutraceutical quality of the extracted oils, the concentrations of two key bioactive compounds—α-tocopherol and γ-oryzanol—were quantified (Table 4). The collected data confirm the consistent presence of significant bioactive compounds across all oil samples, regardless of the extraction method used. Specifically, α-tocopherol levels exceeded 60 µg/g in all samples, with values of 77.14 µg/g for RBO-E1, 69.39 µg/g for RBO-E2, and 116.90 µg/g for RBO-E3. Similarly, γ-oryzanol concentrations were consistently high, averaging around 11 mg/g, equivalent to approximately 1.6% *w*/*w*.

These values are broadly consistent with those reported in the literature, where α-tocopherol concentrations in rice bran oil range between 160 and 460 µg/g [49,50] and γ-oryzanol levels typically vary from around 9.8 mg/g [51] to 17.5 mg/g [52]. It is important to note, however, that these concentrations are affected not only by the extraction technique and storage conditions but also by biological factors, including rice cultivar, cultivation practices, harvest timing, and seasonal climate [53].

Among the tested methods, RBO-E3 once again stood out, with the highest levels of RBO bioactives—116.97 µg/g of α-tocopherol and 13.24 mg/g of γ-oryzanol—compared to RBO-E1 (77.14 µg/g and 11.55 mg/g, respectively) and RBO-E2 (69.39 µg/g and 11.23 mg/g). This improved preservation is likely due to the oxygen- and light-free environment of the SC-CO_2_ extraction process, which helps prevent oxidative degradation of thermolabile compounds [54]. Although differences in α-tocopherol concentrations were not statistically significant, γ-oryzanol levels measured by HPLC were significantly higher in RBO-E3, further supporting the advantage of this green extraction method. In contrast, the lower concentrations observed in RBO-E1 and RBO-E2 may be attributed to exposure to heat and light—during continuous reflux in Soxhlet extraction and during solvent evaporation by rotary evaporator in hexane removal—both of which can accelerate the degradation of sensitive compounds.

### 3.2. Environmental Impact of Rice Bran Oil Extraction Processes

The valorization of rice-processing by-products through oil extraction serves a dual purpose: minimizing agro-industrial waste and recovering valuable bioactive compounds such as γ-oryzanol and tocopherols, which possess significant nutritional and health-promoting properties [18,40]. Nevertheless, the environmental sustainability of different extraction techniques requires a comprehensive evaluation to confirm that ecological advantages are not compromised by high resource consumption or energy demands. LCA emerges as a robust analytical tool to systematically quantify environmental impacts and compare the sustainability profiles of conventional and cutting-edge extraction methods [27,55].

**Figure 2:**
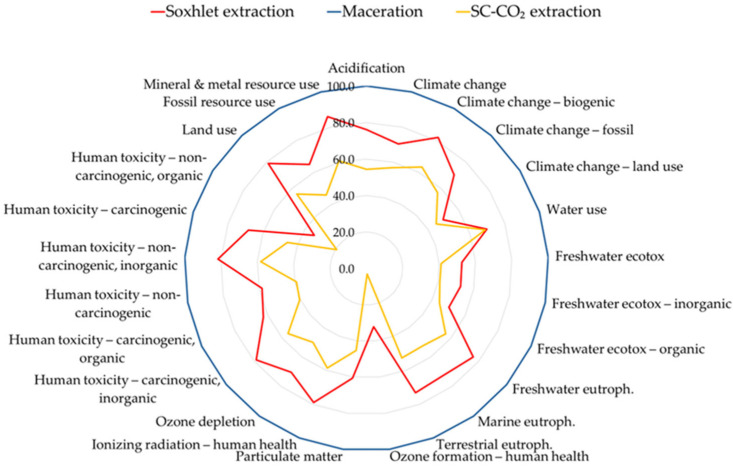
Environmental Impact Profile of the three extraction methods (Case A, FU: 10 g of extracted rice bran oil), assessed according to the EF3.1 method (adapted) and normalized. The worst case represents 100%, and the other values are scaled relative to it (radar chart).

In the present study three extraction routes were evaluated using a cradle- to-gate LCA analysis: Soxhlet extraction (E1), maceration (E2), and supercritical CO_2_ extraction (E3). When results were normalized to 10 g of extracted oil (Case A), E2 consistently exhibited the highest environmental impacts across categories such as climate change, fossil resource use, and toxicity, largely due to high solvent consumption and relatively low yields (Figure 2) [27,56]. E1 presented intermediate values, reflecting its higher extraction efficiency compared to E2 but also its intensive energy requirements. Conversely, E3 showed the lowest environmental footprint overall, confirming the advantages of solvent-free operation and the inert nature of supercritical CO_2_, which minimizes emissions and process losses [55,56].

**Figure 3:**
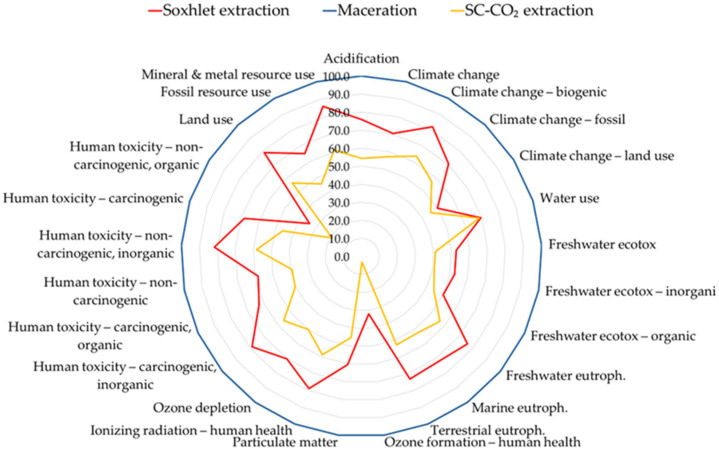
Environmental Impact Profile of the three extraction methods (Case B, FU:1000 μg of alpha-tocopherol), evaluated according to the EF3.1 method (adapted) and normalized. The worst case represents 100% and the other values are scaled with respect to this (radar chart).

When the functional unit was shifted to 1000 µg of α-tocopherol (Case B), the environmental superiority of E3 became even more evident (Figure 3). Since this process achieved the highest α-tocopherol yield (116.9 µg/g oil), the impacts per unit of bioactive compound were substantially lower than those of E1 (77.1 µg/g) and E2 (69.4 µg/g). This observation highlights the importance of selecting functional units that reflect not only extraction efficiency but also the nutritional and functional value of the final product. A similar trend has been reported by Xie et al. [57], who showed that rice bran oil extracted via SC-CO_2_ revealed improved stability and a higher concentration of unsaponifiable compounds compared to solvent-based methods, indicating a better quality-to-impact ratio.

Beyond the assessment of extraction efficiency, LCA interpretation critically depends on the definition of system boundaries. It is recognized that capital goods (e.g., specialized machinery for SC-CO_2_ systems) can contribute significantly to cumulative environmental impacts through manufacturing, material use, and end-of-life phases. Consequently, their inclusion is generally recommended by ISO 14040/44 standards [58]. However, for the scope of this preliminary assessment, the environmental burden associated with the manufacturing of extraction equipment was intentionally excluded. This methodological choice was primarily justified by two practical constraints: the high data uncertainty and limited availability of reliable, process-specific information concerning equipment production, and the difficulty of accurately allocating the long-term environmental burden of such capital-intensive machinery within the limited scope of the present study.

This exclusion is consistent with established practices in the LCA literature, where several comparable studies similarly omit the environmental load of capital goods for these pragmatic reasons [25,55,56]. Accordingly, the system boundaries were strictly focused on stages characterized by robust and traceable data: by-product generation (including rice cultivation), solvent production, and process energy consumption. This approach ensures methodological transparency and enhances comparability with existing literature.

Taken together, these findings align with the growing body of literature on green extraction, where SC-CO_2_ has been shown to outperform conventional solvent extraction both in terms of process sustainability and product quality [30,59]. Above all, the choice of the functional unit strongly affects the ranking of processes: while yield-based comparisons emphasize efficiency, bioactivity-based metrics better capture the nutritional and economic relevance of the recovered compounds. This dual perspective is crucial in the context of circular economy strategies, where the aim is not only to minimize waste but also to maximize the value recovered from by-products [27,48].

To our knowledge, few studies have applied LCA to rice bran oil extraction, and even fewer have explicitly linked environmental performance with bioactive compound yield. However, parallels can be drawn with recent LCA applications in the valorization of tomato pomace [27] and other agri-food by-products, where SC-CO_2_ and microwave-assisted techniques were shown to offer lower environmental footprints compared to conventional extractions. Taken together, these results underscore the relevance of LCA as a decision-making tool to guide the scaling-up of innovative extraction methods. Specifically, the data obtained in this study support the view that SC-CO_2_ extraction represents the most sustainable pathway for rice bran valorization, combining higher product quality with reduced environmental burden.

## 4. Conclusions

The present study proves that rice bran oil, particularly when obtained via supercritical CO_2_ extraction, represents a high-value by-product with significant potential as a natural antioxidant in both food and cosmetic applications. Oil yields were comparable across methods, but SC-CO_2_ extraction maximized the preservation of bioactive compounds, achieving the highest α-tocopherol concentration (116.97 µg/g) and γ-oryzanol content (13.24 mg/g). Antioxidant assays confirmed a superior radical-scavenging activity for E3, with ABTS (6.89 mg TE/g), DPPH (3.84 mg TE/g), and FRAP (6.22 mg TE/g) values all surpassing conventional methods. The fatty acid profile of SC-CO_2_ oil was enriched in unsaturated fatty acids (86.3% total UFA), mainly oleic (C18:1, 55.6%) and linoleic (C18:2, 30.6%) acids, with a low proportion of saturated fraction (13.6%). Free fatty acid analysis revealed minimal hydrolysis, indicating high oil stability, whereas Soxhlet- and maceration-extracted oils showed higher saturated fractions (56.5% and 60.1%, respectively) and elevated free fatty acid levels, reflecting thermal and solvent-induced hydrolysis. Moreover, environmental assessment revealed that SC-CO_2_ extraction exhibited the lowest impacts per functional unit, both per 10 g of oil and per 1000 µg of α-tocopherol, demonstrating the alignment of product quality and sustainability. Collectively, these findings display the potential to valorize rice bran oil as a nutraceutical and functional ingredient, underscoring both its economic and environmental advantages.

## Figures and Tables

**Table 1:** Acidity and peroxide values of extracted rice bran oils.

	AV	PV
	% Oleic Acid	mEq O_2_/kg
RBO-E1	44.22 ± 6.46 ^b^	10.43 ± 2.67 ^a^
RBO-E2	51.17 ± 2.02 ^b^	8.30 ± 2.01 ^a^
RBO-E3	60.14 ± 0.76 ^a^	5.84 ± 0.67 ^a^

RBO-E1: rice bran oil extracted using Soxhlet extraction; RBO-E2: rice bran oil extracted using maceration; RBO-E3: rice bran oil extracted using supercritical CO_2_ extraction; AV: Acidity value; PV: Peroxide value. Values are expressed as mean ± standard deviation. based on three independent biological replicates. each analyzed in triplicate. Data with different superscript letters within the same column differ significantly according to Tukey’s test at *p* < 0.05.

**Table 2:** Fatty-acid methyl ester (FAME) profile of rice bran oils obtained via different extraction methods: individual fatty acids and sums of SFA, MUFA, and PUFA.

IUPAC Name	Common Name	R.T. (min)	RBO-E1 (%)	RBO-E2 (%)	RBO-E3 (%)
Dodecanoic acid, methyl ester	Lauric acid (C12:0)	14.77	ND	0.29	0.05
Methyl tetradecanoate	Myristic acid (C14:0)	19.17	3.88	1.91	0.54
Hexadecanoic acid, methyl ester	Palmitic acid (C16:0)	23.70	25.76	48.10	10.24
Heptadecanoic acid, methyl ester	Margaric acid(C17:0)	25.91	4.56	0.00	0.18
Methyl stearate	Stearic acid (C18:0)	28.07	22.27	6.38	2.09
9-Octadecenoic acid (Z)-, methyl ester	Oleic acid (C18:1)	28.59	43.53	32.35	55.56
9,12-Octadecadienoic acid (Z,Z)-, methyl ester	Linoleic acid (C18:2)	29.63	ND	6.41	30.62
9,12,15-Octadecatrienoic acid, methyl ester, (Z,Z,Z)-	Linolenic acid (C18:3)	31.03	ND	0.66	0.15
Eicosanoic acid, methyl ester	Arachidic acid (C20:0)	32.20	ND	1.57	0.46
11-Eicosenoic acid, methyl ester	Gondoic acid (C20:1)	32.70	ND	0.49	0.11
Docosanoic acid, methyl ester	Behenic acid (C22:0)	36.08	ND	1.84	ND
∑ Saturated Fatty Acid			56.47	60.09	13.56
∑ Monounsaturated Fatty Acid			43.53	32.84	55.67
∑ Polyunsaturated Fatty Acid			0	7.07	30.77

RBO-E1: rice bran oil extracted using Soxhlet extraction; RBO-E2: rice bran oil extracted using maceration; RBO-E3: rice bran oil extracted using supercritical CO_2_ extraction; R.T.: Retention time; ND: Not detected.

**Table 3:** Phenolic compounds and antioxidant activity of extracted rice bran oils.

	TPC	TFC	ABTS	FRAP	DPPH
	mg GAE/g	mg CE/g	mg TE/g	mg TE/g	mg TE/g
RBO-E1	1.76 ± 0.34 ^a^	4.67 ± 0.25 ^b^	5.25 ± 0.54 ^b^	3.03 ± 1.03 ^b^	3.01 ± 0.38 ^b^
RBO-E2	1.91 ± 0.33 ^a^	5.52 ± 0.48 ^a^	6.18 ± 1.31 ^ab^	4.79 ± 0.57 ^a^	3.64 ± 0.55 ^a^
RBO-E3	2.05 ± 0.12 ^a^	5.46 ± 0.37 ^a^	6.89 ± 0.42 ^a^	6.22 ± 0.36 ^a^	3.84 ± 0.10 ^a^

RBO-E1: rice bran oil extracted using Soxhlet extraction; RBO-E2: rice bran oil extracted using maceration; RBO-E3: rice bran oil extracted using supercritical CO_2_ extraction; TPC: Total Phenolic Content; TFC: Total Flavonoid Content; FRAP: Ferric Reducing Antioxidant Power; ABTS: 2,2′-Azino-bis(3-ethylbenzothiazoline-6-sulfonic acid) radical scavenging activity; DPPH: 2,2-Diphenyl-1-picrylhydrazyl radical scavenging activity. Values are expressed as mean ± standard deviation, based on three independent biological replicates, each analyzed in triplicate. Data with different superscript letters within the same column differ significantly according to Tukey’s test at *p* < 0.05.

**Table 4:** Alpha-tocopherol and γ-oryzanol content in rice bran oils obtained via different extraction methods.

	α-Tocopherol	γ-Oryzanol
	µg/g	mg/g
RBO-E1	77.14 ± 14.47 ^a^	11.55 ± 0.26 ^b^
RBO-E2	69.39 ± 12.14 ^a^	11.23 ± 0.14 ^c^
RBO-E3	116.90 ± 9.14 ^b^	13.24 ± 0.04 ^a^

RBO-E1: rice bran oil extracted using Soxhlet extraction; RBO-E2: rice bran oil extracted using maceration; RBO-E3: rice bran oil extracted using supercritical CO_2_ extraction. Values are expressed as mean ± standard deviation, based on three independent biological replicates, each analyzed in triplicate. Data with different superscript letters within the same column differ significantly according to Tukey’s test at *p* < 0.05.

## Data Availability

The original contributions presented in the study are included in the article, further inquiries can be directed to the corresponding author.
